# The NIPH’s response to the climate and environmental crisis

**DOI:** 10.1093/eurpub/ckac129.226

**Published:** 2022-10-25

**Authors:** A Aakra

**Affiliations:** Division for Climate and Environmental Health, Norwegian Institute of Public Health, Oslo, Norway

## Abstract

The vision of the Norwegian Institute of Public Health (NIPH) is better health for all. Climate changes and health are among our main strategic priorities. The NIPH aims to become a key actor in questions relating to climate, environment, food and health. An action plan on climate changes and environmental health is under development and will be presented. Climate and environmental changes affect the mental and physical health in several ways. The NIPH will in the coming years develop new knowledge on health consequences of climate changes: causes, risks, health effects and measures, based on our methodological expertise, research activities and infrastructures, such as health registers, health examinations, laboratories and biobanks. A key issue, and a main priority of the NIPH, is sustainable and climate-adapted food systems. There is a strong need to strengthen knowledge on healthy, safe and globally sustainable food. We will present and discuss our action plan for addressing climate and environmental health: Priorities, experiences, and challenges in implementation regarding e.g. competence and capacity building.

The following examples will be given:

– Research that addresses the food problem, and how to ensure a sustainable diet.

– The development of the MoBa Human Environmental Biobank to understand how climate and environmental changes affect exposure and public health, and how to follow the changes over time and help prevent disease.

– Examples of activities relating to clean air, clean and safe drinking water, and safe products from a One Health perspective: Achievements, projects, networks.

– Challenges in communicating health risks associated with the climate, environmental and pollution crisis to the public.

We encourage feedback and suggestions on NIPH's efforts to address the climate crisis, to ensure good knowledge sharing and that we are fully supporting our stakeholders.

